# Coronavirus disease (COVID-19): a scoping review

**DOI:** 10.2807/1560-7917.ES.2020.25.15.2000125

**Published:** 2020-04-16

**Authors:** Meng Lv, Xufei Luo, Janne Estill, Yunlan Liu, Mengjuan Ren, Jianjian Wang, Qi Wang, Siya Zhao, Xiaohui Wang, Shu Yang, Xixi Feng, Weiguo Li, Enmei Liu, Xianzhuo Zhang, Ling Wang, Qi Zhou, Wenbo Meng, Xiaolong Qi, Yangqin Xun, Xuan Yu, Yaolong Chen, on behalf of the COVID-19 evidence and recommendations working group

**Affiliations:** 1 School of Public Health, Lanzhou University, Lanzhou, China; 2 These authors contributed equally to this work and share first authorship; 3 Evidence-based Medicine Center, School of Basic Medical Sciences, Lanzhou University, Lanzhou, China; 4 Institute of Global Health, University of Geneva, Geneva, Switzerland; 5 Institute of Mathematical Statistics and Actuarial Science, University of Bern, Bern, Switzerland; 6 Department of Health Research Methods, Evidence and Impact, Faculty of Health Sciences, McMaster University, Hamilton, Canada; 7 College of Medical Information Engineering, Chengdu University of Traditional Chinese Medicine, Chengdu, China; 8 School of Public Health, Chengdu Medical College, Chengdu, China; 9 Department of Respiratory Diseases, Children’s Hospital of Chongqing Medical University, Chongqing, China; 10 Chongqing Key Laboratory of Pediatrics, Chongqing, China; 11 The First School of Clinical Medicine, Lanzhou University, Lanzhou, China; 12 The First Hospital of Lanzhou University, Lanzhou, China; 13 World Health Organization (WHO) Collaborating Centre for Guideline Implementation and Knowledge Translation, Lanzhou, China; 14 Guideline International Network Asia, Lanzhou, China; 15 Key Laboratory of Evidence Based Medicine and Knowledge Translation of Gansu Province, Lanzhou University, Lanzhou, China; 16 Lanzhou University, an affiliate of the Cochrane China Network, Lanzhou, China; 17 The study collaborators are acknowledged at the end of the article

**Keywords:** COVID-19, SARS-CoV-2, scoping review, communicable diseases, pandemics, coronavirus infections, global health emergency

## Abstract

**Background:**

In December 2019, a pneumonia caused by a novel coronavirus (SARS-CoV-2) emerged in Wuhan, China and has rapidly spread around the world since then.

**Aim:**

This study aims to understand the research gaps related to COVID-19 and propose recommendations for future research.

**Methods:**

We undertook a scoping review of COVID-19, comprehensively searching databases and other sources to identify literature on COVID-19 between 1 December 2019 and 6 February 2020. We analysed the sources, publication date, type and topic of the retrieved articles/studies.

**Results:**

We included 249 articles in this scoping review. More than half (59.0%) were conducted in China. Guidance/guidelines and consensuses statements (n = 56; 22.5%) were the most common. Most (n = 192; 77.1%) articles were published in peer-reviewed journals, 35 (14.1%) on preprint servers and 22 (8.8%) posted online. Ten genetic studies (4.0%) focused on the origin of SARS-CoV-2 while the topics of molecular studies varied. Nine of 22 epidemiological studies focused on estimating the basic reproduction number of COVID-19 infection (R_0_). Of all identified guidance/guidelines (n = 35), only ten fulfilled the strict principles of evidence-based practice. The number of articles published per day increased rapidly until the end of January.

**Conclusion:**

The number of articles on COVID-19 steadily increased before 6 February 2020. However, they lack diversity and are almost non-existent in some study fields, such as clinical research. The findings suggest that evidence for the development of clinical practice guidelines and public health policies will be improved when more results from clinical research becomes available.

## Introduction

A new type of coronavirus (severe acute respiratory syndrome coronavirus 2; SARS-CoV-2) that began in Wuhan, China in late 2019 has spread across the world since then. The virus has caused an outbreak of viral pneumonia, which has been named Coronavirus disease (COVID-19). As of 24:00 on 6 February 2020, over 31,000 cases and 636 deaths had been confirmed in China [1]. Furthermore, more than 1,770,000 cases had been diagnosed in 213 countries, areas or territories as at 13 April 2020 [2]. On 23 January 2020, Chinese authorities imposed a lockdown of Wuhan [3]. On 30 January 2020, the World Health Organization (WHO) declared the outbreak a Public Health Emergency of International Concern (PHEIC) [4] and on 11 March 2020, a pandemic [5].

The WHO [6-9], the United States (US) Centers for Disease Control and Prevention (CDC) [10,11], the European Centre for Disease Prevention and Control (ECDC) [12,13] as well as Chinese researchers have issued several guidance documents or guidelines to help address the outbreaks. Meanwhile, many scientific journals have rapidly published a number of articles, comments, editorials and perspectives related to COVID-19. It may however be challenging for the global research community to find all the available evidence: many of the first studies on COVID-19 were published in Chinese, and because of the rapidly developing situation, the latest studies are often available on websites or preprint servers only [14].

Scoping reviews are regarded as a valid tool to map the available evidence on a given topic, to clarify the characteristics of body of literature, to organise the key concepts and their relationship and to analyse knowledge gaps [15]. The methodology continues to be developed, and a Preferred Reporting Items for Systematic Reviews and Meta-Analyses (PRSIMA) extension for Scoping Reviews (PRISMA-SCR) including reporting guidance was published in 2018 [16]. Given the urgency of the COVID-19 epidemic and the need to understand and access information about it, a scoping review was considered suitable for the situation. We therefore conducted this scoping review to help identify research gaps related to this new viral disease and propose recommendations for future research on COVID-19.

## Methods

### Search strategy

We performed a systematic search of MEDLINE via PubMed, Embase, Web of Science, China National Knowledge Infrastructure (CNKI), Wanfang Data and China Biology Medicine (CBM) on 27 February 2020 with the terms “COVID-19” OR “SARS-CoV-2” OR “2019 novel coronavirus” OR “2019-nCoV” OR “Wuhan coronavirus” OR “novel coronavirus” OR “Wuhan seafood market pneumonia virus” OR “Wuhan virus”, published between 1 December 2019 and 6 February 2020 (see Supplement S1 for details of search strategies). Because of potential delays in indexing of databases, we also searched selected infectious disease journals (Supplementary Table S1). We also searched Google Scholar; the official websites of WHO (https://www.who.int/), US CDC (https://www.cdc.gov/), ECDC (https://www.ecdc.europa.eu/en), Public Health England (PHE) (https://www.gov.uk/government/organisations/public-health-england); some preprint servers, including BioRxiv (https://www.biorxiv.org/), ChemRxiv (https://chemrxiv.org/), medRxiv (https://www.medrxiv.org/) and SSRN (https://www.ssrn.com/index.cfm/en/); and reference lists of the identified articles to find reports of additional studies.

### Inclusion and exclusion criteria

We included all literature related to COVID-19 published in English and Chinese between 1 December 2019 and 6 February 2020 without restrictions, including guidance/guidelines, reviews, clinical studies, basic research, epidemiological studies and comments. Documents and guidance/guidelines posted by international organisations, government institutions, associations and societies were also included. We excluded news reports that were not published in scientific journals, and articles where we failed to access full text despite contacting the authors.

### Article selection and data extraction

Two reviewers (ML and XL) screened all titles, abstracts and full texts independently and solved disagreements by consensus or consultation with a third reviewer. Then the following information was extracted: (i) title, (ii) first author, (iii) whether peer-reviewed or not, (iv) journal, (v) publication or posted date, (vi) first author’s country (or international organisation), (vii) type of article/study and (viii) topic. The details are shown in Supplementary Table S2.

### Data analysis

We conducted a descriptive analysis of the characteristics of the included literature. We described the source where we found the article, publication date, type of article/study, and topic of article/study or guidance/guideline on COVID-19 to examine the existing gaps in research. We categorised the literature into guidance/guidelines and consensus statements, reviews, clinical studies (including randomised controlled trials and observational studies), basic research, epidemiological studies, editorial comments on COVID-19 and other categories if identified. We conducted this scoping review in accordance with the PRISMA-ScR Checklist [16] (Supplementary Table S3).

## Results

### Search results

**Figure 1:**
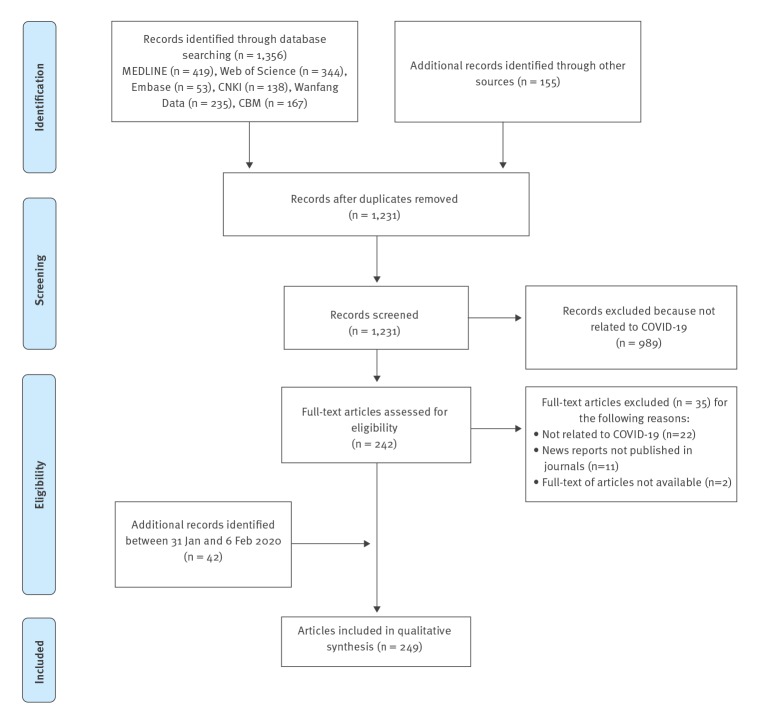
Flowchart of selection process for the scoping review of coronavirus disease (COVID-19) articles/studies and results, 1 December 2019–6 February 2020

CBM: China Biology Medicine; CNKI: China National Knowledge Infrastructure.

We identified 1,511 records, 280 of which were excluded as duplicates. Title and abstract screening were conducted for the remaining 1,231 articles, 989 of which were excluded because of being unrelated to COVID-19. For two articles, we failed to access the full text after contacting the authors. We retrieved the full texts of the 242 remaining articles. After further screening and supplementary searching of articles published or posted between 31 January 2020 and 6 February 2020, we identified an additional 42 articles and a total of 249 articles were included in the review (Figure 1).

### Characteristics of included articles/studies

Of the 249 included articles/studies, 147 (59.0%) were from China. The article/study type varied vastly, which we broadly characterised into 11 types (Table 1). Of these, guidance/guidelines and consensuses statements were the most common (n = 56; 22.5%).

**Table 1:** Characteristics of the coronavirus disease (COVID-19) articles/studies included in the scoping review, 10 January–6 February 2020 (n = 249)

Characteristic		Number of articles/studies	Percentage (%)
Publication platform	Journal	192	77.1
	Other than journal^a^	57	22.9
Journal (n = 192)	*The Lancet*	13	6.8
	*Journal of Medical Virology*	12	6.3
	*New Medicine*	9	4.7
	*The New England Journal of Medicine*	9	4.7
	*Eurosurveillance*	8	4.2
	*Journal of Traditional Chinese Medicine*	7	3.6
	*British Medical Journal (BMJ)*	7	3.6
	*Radiology*	5	2.6
	*Travel Medicine and Infectious Disease*	5	2.6
	*Chinese Nursing Research*	5	2.6
	*Chinese Journal of Tuberculosis and Respiration*	4	2.1
	*Nature*	4	2.1
	*Chinese Journal of Contemporary Paediatrics*	3	1.6
	*Emerging Microbes and Infections*	3	1.6
	*The Journal of the American Medical Association (JAMA)*	3	1.6
	*Journal of Hospital Infection*	3	1.6
	*Journal of Travel Medicine*	3	1.6
	*Herald of Medicine*	3	1.6
	*Chinese Journal of Emergency Medicine*	3	1.6
	*Chinese Journal of Paediatrics*	3	1.6
	Other	80	41.7
First author’s country or international organisation	China	147	59.0
	United States	33	13.3
	United Kingdom	16	6.4
	WHO	10	4.0
	Canada	7	2.8
	Germany	6	2.4
	Other	30	12.1
Publication or posted date	10–15 Jan	6	2.4
	16–20 Jan	7	2.8
	21–25 Jan	38	15.3
	26–31 Jan	93	37.3
	1–6 Feb	105	42.2
Type of article/study	Guidance/guideline or consensus statement	56	22.6
	Review	39	15.7
	Basic research	35	14.1
	Letter	25	10.0
	Epidemiological study^b^	22	8.8
	Editorial	20	8.0
	Comments	11	4.4
	News item	9	3.6
	Case report	9	3.6
	Cross-sectional study	7	2.8
	Case series	5	2.0
	Other	11	4.4
Topic	Prevention and control	33	13.3
	Outbreak reporting	30	12.0
	Genetics	22	8.8
	Transmissibility	22	8.8
	Clinical features	21	8.4
	Diagnosis and treatment	19	7.6
	Molecular biology	15	6.0
	Management	14	5.6
	Characteristics of SARS-CoV-2^c^	11	4.4
	Drug-related^d^	8	3.2
	Traditional Chinese medicine	8	3.2
	Lessons and challenges	7	2.8
	Transmission pattern	7	2.8
	Surveillance and screening	5	2.0
	Mental health	4	1.6
	Other	23	9.2

SARS-CoV-2: severe acute respiratory syndrome coronavirus 2; WHO: World Health Organization.

^a^ Includes the websites of WHO, United States Centers for Disease Control and Prevention (US CDC), European Centre for Disease Prevention and Control (ECDC) and Public Heath England (PHE), and preprint servers.

^b^ Other than cross-sectional studies.

^c^ Includes reviews and correspondence that discussed the characteristics of the virus in general.

^d^ Other than traditional Chinese medicine.

### Sources of articles/studies

Of all included articles/studies, 192 (77.1%) were published in peer-reviewed journals, 35 (14.1%) were posted on preprint servers and 22 (8.8%) were published on the official websites of public health organisations. The journal with the highest number of articles was The Lancet, with 13 (6.8%) published articles. Of preprint articles, most (n = 28) were posted on BioRxiv. Articles published on official websites were mainly COVID-19 guidance/guidelines, including 10 WHO interim guidance documents, nine US CDC interim guidelines/guidance documents, two ECDC guidance documents and one Communicable Diseases Network Australia (CNDA) guideline.

### Publication date

**Figure 2:**
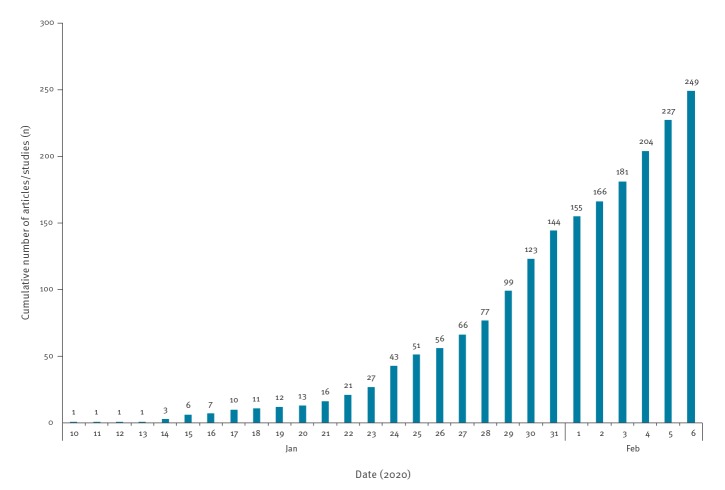
Cumulative number of coronavirus disease (COVID-19)-related articles/studies included in the scoping review, 10 January–6 February 2020 (n = 249)

Figure 2 shows the cumulative number of articles published daily between 10 January 2020 and 6 February 2020. As at 6 February 2020, the number of articles on COVID-19 had been steadily increasing. Of the 192 articles that were published in peer-reviewed journals, the highest number of journal publications on a single day was on 30 January, with 24 articles (12.5%). For the 35 preprints, the number posted per day rose steadily from 19 January 2020 to 6 February 2020.

### Type of article/study

**Figure 3:**
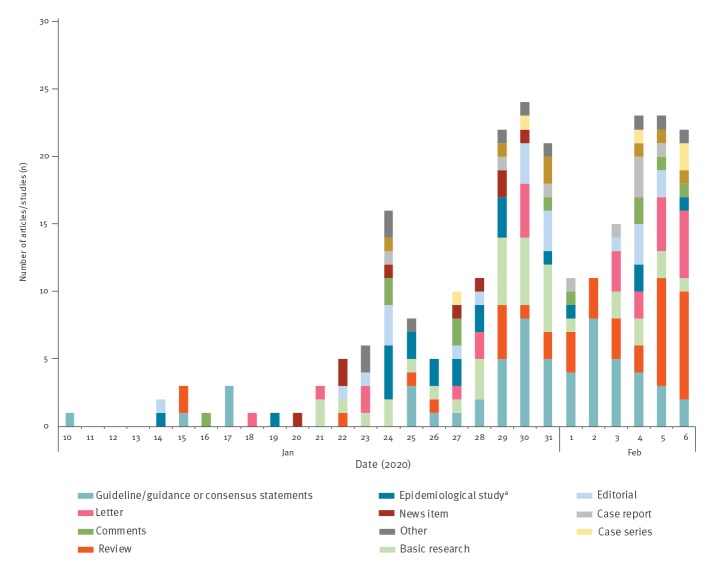
Number of coronavirus disease (COVID-19)-related articles/studies published per day according to type, 10 January–6 February 2020 (n = 249)

^a^ Including cross-sectional studies.

The types of articles/studies published on each day are shown in Figure 3. The daily number of guidance/guidelines peaked between 29 January and 3 February whereas the number of published reviews showed an increasing trend since 29 January 2020. Only one systematic review was identified [17]. We found no randomised controlled studies or cohort studies.

### Topics

The different types of articles/studies focused on different topics. The basic research could be divided broadly into two categories: 21 genetic studies and 12 molecular biology studies. Ten genetic studies traced the origin of SARS-CoV-2 and tried to determine the possible virus reservoir. Among these, most suggested that SARS-CoV-2 evolved from a bat-CoV, namely bat-SL-CoVZC45, bat-SL-CoVZXC21, bat-SL-CoVZX45 and bat-CoV-RaTG13 as potential candidates [18-26]. However, Ji et al. [18] found snakes to be the most probable reservoir for SARS-CoV-2 while Guo et al. [26] suggested mink could be a candidate reservoir. Of the molecular studies, five [27-31] showed that the key receptor of SARS-CoV-2 is angiotensin converting enzyme 2 (ACE2), which is highly expressed in lung type II alveolar cells (AT2) [27], positive cholangiocytes [29], upper oesophagus, stratified epithelial cells and absorptive enterocytes from ileum and colon [30]. The other studies included an assessment of the cross-reactivity of anti-SARS-CoV antibodies with SARS-CoV-2 spike protein [32], and SARS-CoV-2 main proteases [33,34].

The main topic of epidemiological studies was the estimation of the transmissibility of COVID-19. The value of the basic reproduction number (R_0_) varied across studies [35-43], however, all estimated it to be higher than one, which indicates the potential for sustained human-to-human transmission. According to the nine articles [35-43], R_0_ ranges between 2.2 and 3.9. Some studies showed that the transmissibility of SARS-CoV-2 is comparable to [37,44] or even higher [39] than SARS-CoV and Middle East respiratory syndrome coronavirus (MERS-CoV). In addition, studies focused on the disease burden associated with COVID-19 [45] and the global patterns of disease dispersion [46,47].

Most reviews on COVID-19 gave a brief summary of the clinical features [48-51] and the characteristics of SARS-CoV-2 [52-54], as well as recommendations on how to prevent and control [55-60] this novel pneumonia. A systematic review [17] explored the possibility of using lopinavir/ritonavir (LPV/r) to treat COVID-19, with the results supporting the use of LPV/r as a part of an experimental regimen for COVID-19 pneumonia treatment. Clinical features were reported in 21 studies [48-51,61-77]. The main symptoms of patients with COVID-19 at onset were found to be fever and cough, with a reduced lymphocyte count, which is similar to previous beta coronavirus infections [78,79].

Seventeen of the 56 editorials, comments and letters [80-96] were first reports or comments on the situation of the COVID-19 epidemic. Some [97-101] also briefly introduced the general information and characteristics of the new virus. The mapping of article/study type and topics, as well as associated gaps, is shown in Table 2.

**Table 2:** Mapping of coronavirus disease (COVID-19) article/study types and topics, 10 January–6 February 2020 (n = 249)

Topic	Article type											
	Guidance/guideline or consensus statement (n)	Review (n)	Basic research (n)	Letter (n)	Epidemiological study^a^ (n)	Editorial (n)	Comments (n)	News item (n)	Case report (n)	Cross-sectional study (n)	Case series (n)	Other^b^ (n)
Prevention and control	23	6	0	2	2	0	0	0	0	0	0	0
Outbreak reporting	0	0	0	3	0	10	4	9	0	0	0	4
Genetics	0	1	21		0	0	0	0	0	0	0	0
Transmissibility	0	1	0	4	13	3	0	0	0	0	1	0
Clinical features	0	4	0	2	0	0	2	0	5	2	4	2
Diagnosis and treatment	11	3	0	1	0	1	0	0	2	0	0	1
Molecular biology	0	2	12	1	0	0	0	0	0	0	0	0
Management	12	2	0		0	0	0	0	0	0	0	0
Characteristics of SARS-CoV-2	0	4	0	1	0	3	1	0	1	0	0	1
Drug-related^c^	0	2	2	3	0	0	0	0	0	0	0	1
Traditional Chinese medicine	0	8	0		0	0	0	0	0	0	0	0
Lessons and challenges	0	3	0	1	0	0	3	0	0	0	0	0
Transmission pattern	0	0	0	2	4	0	0	0	1	0	0	0
Surveillance and screening	2	0	0	3	0	0	0	0	0	0	0	0
Mental health	0	0	0	1	0	0	1	0	0	2	0	0
Other^d^	8	3	0	1	3	3	0	0	0	3	0	2

^a^ Other than cross-sectional studies.

^b ^Includes perspectives, case-control study and investigation protocols.

^c^ Other than traditional Chinese medicine.

^d^ Guidance/guideline or consensus statement: guidance for laboratory biosafety, caring and travellers, and national capacity review tools; review: reviews on human resources of healthcare, the causes and counter-measures of Wuhan ‘stigma’, and public health; letter: outbreak assessment; epidemiology study: studies on disease burden, the number of unreported cases, and infection fatality; editorial: journal’s opinion on matters related to COVID-19, and incidence rate estimation; cross-sectional study: hazard vulnerability analyses, epidemiology reports, and studies on public attitudes and perception; other: investigation protocol.

### Guidance/guidelines and consensus statements

Of the 56 published guidance/guidelines and consensuses statements, 35 were guidance/guidelines. Nine of the 35 addressed the treatment and management of COVID-19 infection, eight addressed prevention and five addressed diagnostics. Ten of the guidance/guidelines were interim guidance documents issued by the WHO, including those on COVID-19 prevention, surveillance, assessment, care, management and mask use [6-9,102-107]. The US CDC published nine interim guidance/guidelines documents for evaluating, preventing and managing the new coronavirus [10,11,108-114]. In addition, ECDC published two guidance documents about COVID-19 patient care and the management of persons having had contact with SARS-CoV-2 cases [12,13]. Chinese researches also published 14 rapid-advice guidance/guidelines documents on diagnosis, prevention and management of COVID-19, all of which were interim guidance/guidelines documents developed by hospitals [115-128].

Only eight of the guidance documents/guidelines formed a guideline development group (GDG) [129]; the recommendations of 15 guidance documents/guidelines, including six developed by the WHO, were difficult to distinguish. Only ten guidance/guidelines fulfilled the strict principles of evidence-based practice and cited reference documents, which were mainly epidemic reports, government documents, and indirect evidence related to SARS-CoV or MERS-CoV [6,7,105,116-118,120,122,125,126]. Only two guidelines, both developed by Chinese researchers, were graded using the Grading of Recommendations Assessment, Development and Evaluation (GRADE) approach [116,117]. Among the 35 guidance/guidelines, one [115] was completely on Traditional Chinese medicine and one [116] covered Chinese medicine. One Australian guideline [130] was adapted from SARS-CoV guidelines.

## Discussion

Our scoping review shows that while the number of articles on COVID-19 has been constantly increasing, as at 6 February, there were still clear gaps in several study types and research fields. We identified that some study types, in particular randomised controlled trials and cohort studies, were still non-existent before 6 February. According to a preliminary search of the Cochrane Network database up to 10 April 2020, the number of randomised controlled trials (RCTs) (n = 8) and observational studies (n = 42) still remains low [131].

We also found that there were only a few studies on clinical practice, making it difficult to develop clinical practice guidelines and health policies. The reason for the gaps in this area may be the rapid development of the outbreak and limited understanding of the new virus and the disease caused by it. Moreover, it takes time to conduct clinical research. When facing a public health emergency with a previously unknown cause, researchers should conduct studies on whether some clinical practice and public health interventions from other public health emergencies can be used as indirect evidence. However, we identified no such studies in our review.

We found that 14% of the studies related to COVID-19 were posted on preprint servers. This approach of sharing research as quickly as possible is very reasonable, especially in the case of such public health emergency. Previous studies have shown that preprints can accelerate progress in handling outbreaks of infectious disease [132,133].

The research topics in different types of articles/studies had both similarities and differences. Basic research was mostly focused on exploring the origin and reservoirs of the new virus, while epidemiological studies mainly focused on its transmissibility. Reviews and reports provided more general information of the virus and the outbreak, while guidance/guidelines included recommendations on how to prevent and control it.

Clinical practice guidelines are statements that include recommendations intended to optimise patient care that are informed by a systematic review of evidence and an assessment of the benefits and harms of alternative care options [134]. Clinical practice guidelines can inform healthcare workers' actions [134], and, especially when public health emergencies occur, rapid advice guidelines can guide clinicians in terms of how to perform related work [135]. After the outbreak of COVID-19, the WHO, US CDC and ECDC released guidance/guidelines as soon as possible, as did several Chinese institutions. However, most of these documents did not establish formal guideline development groups, and they did not fulfil the strict principles of evidence-based practice. For example, most guidance/guidelines did not grade the quality of evidence and strength of recommendations, and thus owed to the emerging crisis, such guidance/guidelines need to be considered with these limitations in mind. In 2007, the WHO published guidance about the process of developing rapid advice guidelines [129], stating that when a public health emergency occurs, a rapid review is needed and the development time should not exceed 6 months [135]. However, considering the limited time to set up panels, this could be a challenge for guidance/guideline developers. Nonetheless, we still expect guidance/guideline developers to establish formal development groups and fulfil the evidence-based practice principles.

 Our scoping review can help researchers identify research gaps so as to conduct research to fill these gaps. For example, in the current situation, a systematic review to estimate the incubation period or research on new drugs or treatments, would be of great importance. This scoping review has several strengths. We performed a systematic search of a comprehensive set of sources, including databases, preprint servers, and official websites of international organisations and associations at the early stage of the pandemic. Furthermore, our large sample size is sufficient to illustrate the state of research and identify research gaps related to COVID-19 at the onset of the pandemic.

This study also has some limitations. Because of the delay in indexing, some articles published as at 6 February 2020 may not have been identified. Also, because our retrieval time was only until this date, articles published or posted after this date, of which there have been many, have not been included in the analysis. As some preprints, guidance/guidelines and disease control plans are constantly updated, the publication date we extracted may not be the time of their first publication time. Also, we did not assess the quality of the included literature because of diversity of the types of included articles. Another limitation of our study was that it only included articles published in English and Chinese, which could introduce publication bias. However, as the epidemic was most heavily affecting China until early February, it is reasonable to expect that literature published in English and Chinese up until this point in time covered the majority of the available knowledge. Finally, we were unable to access the full texts of two articles despite contacting the authors. However, compared with the total number of articles included in the review, we anticipate that the exclusion of these two articles is unlikely to have a major impact.

## Conclusion

This scoping review shows the state of literature published or posted online related to COVID-19 as at 6 February 2020. The number of articles in this field has steadily increased since the outbreak became evident. However, the types of studies lacked diversity, especially clinical studies. More clinical research is needed, but in the rapidly evolving global pandemic, we encourage researchers to continuously review the latest literature, to take into account the latest available evidence and avoid overlapping work, and to improve evidence for the development of clinical practice guidelines and public health policies.

## Supplementary Data

164000 Supplementary S1

## Supplementary Data

261000 Supplementary Table S1

## Supplementary Data

59000 Supplementary Table S2

## Supplementary Data

134000 Supplementary Table S3
