# Supplementary material for: Coronavirus disease (COVID-19): a scoping review
Source: Euro Surveill. 2020 Apr 16;25(15):2000125. doi: 10.2807/1560-7917.ES.2020.25.15.2000125 (PMC7175649; doi:10.2807/1560-7917.ES.2020.25.15.2000125)
Supplement: Supplementary S1 [file 20-00125_LV_SupplementS1.pdf]

Supplement S1 Search Strategy

"This supplementary material is hosted by *Eurosurveillance* as supporting information alongside the article [Coronavirus disease (COVID-19): a scoping review], on behalf of the authors, who remain responsible for the accuracy and appropriateness of the content. The same standards for ethics, copyright, attributions and permissions as for the article apply. Supplements are not edited by *Eurosurveillance* and the journal is not responsible for the maintenance of any links or email addresses provided therein."

MEDLINE

#1. ‘Wuhan coronavirus’ [Supplementary Concept]
#2. ‘novel coronavirus’[Title/Abstract]
#3. ‘Wuhan virus’[Title/Abstract]
#4. ‘Wuhan-Cov’[Title/Abstract]
#5. ‘Wuhan seafood market pneumonia virus’[Title/Abstract]
#6. ‘2019-CoV’[Title/Abstract]
#7. ‘2019-nCoV’[Title/Abstract]
#8. ‘2019 novel coronavirus’[Title/Abstract]
#9. ‘COVID-19’ [Supplementary Concept]
#10. ‘Novel CoV’[Title/Abstract]
#11. ‘SARS-CoV-2’[Title/Abstract]
#12. ‘novel coronavirus pneumonia’[Title/Abstract]
#13. ‘severe acute respiratory syndrome coronavirus 2’ [Supplementary Concept]
#14. OR/#1-#13
#15. ‘2019/12/01’[Date - Publication] : ‘2020/02/06’[Date - Publication])
#16. #14 AND #15

Web of Science

#1. Topic: (COVID-19)
#2. Topic: (Wuhan coronavirus)
#3. Topic: (novel coronavirus)
#4. Topic: (Wuhan virus)
#5. Topic: (Wuhan-Cov)
#6. Topic: (Wuhan seafood market pneumonia virus)
#7. Topic: (2019-CoV)
#8. Topic: (2019-nCoV)
#9. Topic: (2019 novel coronavirus)
#10. Topic: (Novel CoV)
#11. Topic: (SARS-CoV-2)
#12. Topic: (novel coronavirus pneumonia)
#13. Topic: (severe acute respiratory syndrome coronavirus 2)
#14. OR/#1-#13
#15. Limted publish year 2020
#16. #14 AND #15

Embase

#1. ' severe acute respiratory syndrome coronavirus 2'/exp

#2. ' COVID-19'/exp

#3. ‘Wuhan coronavirus’ :ab,ti

#4. ‘novel coronavirus’ :ab,ti

#5. ‘Wuhan virus’ :ab,ti

#6. ‘Wuhan-Cov’ :ab,ti

#7. ‘Wuhan seafood market pneumonia virus’ :ab,ti

#8. ‘2019-CoV’ :ab,ti

#9. ‘2019-nCoV’ :ab,ti

#10. ‘2019 novel coronavirus’ :ab,ti

#11. ‘COVID-19’ :ab,ti

#12. ‘Novel CoV’ :ab,ti

#13. ‘SARS-CoV-2’ :ab,ti

#14. ‘novel coronavirus pneumonia’ :ab,ti

#15. ‘severe acute respiratory syndrome coronavirus 2’ :ab,ti

#16. OR/#1-#15

#17. lim 2020

#18. #16 AND #17

#19. #18/medline

#20. #18 NOT #19

CNKI (https://www.cnki.net)

#1. "新型冠状病毒"[主题]

#2. "2019-nCoV"[主题]

#3. "2019-CoV"[主题]

#4. "COVID-19"[主题]

#5. "武汉冠状病毒"[主题]

#6. "SARS-CoV-2"[主题]

#7. "武汉肺炎"[主题]

#8. "新冠肺炎"[主题]

#9. OR/#1-#8

#10. lim 2019.12.1~2020.2.6

#11. #9 AND #10

Wanfang Data (http://www.wanfangdata.com.cn/index.html)

#1. "新型冠状病毒"[主题]

#2. "2019-nCoV"[主题]

#3. "2019-CoV"[主题]

#4. "COVID-19"[主题]

#5. "武汉冠状病毒"[主题]

#6. "SARS-CoV-2"[主题]

#7. "武汉肺炎"[主题]

#8. "新冠肺炎"[主题]

#9. OR/#1-#8

#10. lim 2019.12.1~2020.2.6

#11. #9 AND #10

CBM (http://www.sinomed.ac.cn)

#1. "2019 冠状病毒"[常用字段:智能]

#2. "新型冠状病毒"[常用字段:智能]

#3. "新冠肺炎"[常用字段:智能]

#4. "2019-nCoV"[常用字段:智能]

#5. "SARS-CoV-2"[常用字段:智能]

#6. "Novel coronavirus"[常用字段:智能]

#7. "nCoV"[常用字段:智能]

#8. "Emerging Coronaviruses"[常用字段:智能]

#9. "new coronavirus"[常用字段:智能]

#10. "COVID-19"[常用字段:智能]

#11. "coronavirus"[常用字段:智能]

#12. OR/#1-#11

#13. 2020-2020[日期]

#14. #12 AND #13
