# Supplementary material for: Coronavirus disease (COVID-19): a scoping review
Source: Euro Surveill. 2020 Apr 16;25(15):2000125. doi: 10.2807/1560-7917.ES.2020.25.15.2000125 (PMC7175649; doi:10.2807/1560-7917.ES.2020.25.15.2000125)
Supplement: Supplementary Table S1 [file 20-00125_LV_SupplementaryTableS1.pdf]

Supplementary Table S1 Infectious diseases journals searched

"This supplementary material is hosted by *Eurosurveillance* as supporting information alongside the article [Coronavirus disease (COVID-19): a scoping review], on behalf of the authors, who remain responsible for the accuracy and appropriateness of the content. The same standards for ethics, copyright, attributions and permissions as for the article apply. Supplements are not edited by *Eurosurveillance* and the journal is not responsible for the maintenance of any links or email addresses provided therein."

No.	Journal	URL
1	LANCET INFECTIOUS DISEASES	https://www.thelancet.com/journals/laninf/home
2	CLINICAL INFECTIOUS DISEASES	https://academic.oup.com/cid
3	EUROSURVEILLANCE	https://www.eurosurveillance.org/
4	EMERGING INFECTIOUS DISEASES	https://wwwnc.cdc.gov/eid/
5	CLINICAL MICROBIOLOGY AND INFECTION	https://www.clinicalmicrobiologyandinfection.com/
6	JOURNAL OF ANTIMICROBIAL CHEMOTHERAPY	https://academic.oup.com/jac
7	JOURNAL OF INFECTION	https://www.journalofinfection.com/
8	JOURNAL OF INFECTIOUS DISEASES	https://academic.oup.com/jid
9	ACS INFECTIOUS DISEASES	https://pubs.acs.org/journal/aidcbc
10	TRAVEL MEDICINE AND INFECTIOUS DISEASE	https://www.sciencedirect.com/journal/travel-medicine-and-infectious-disease
11	VIRULENCE	https://www.tandfonline.com/toc/kvir20/current
12	INFECTIOUS DISEASE CLINICS OF NORTH AMERICA	https://www.sciencedirect.com/journal/infectious-disease-clinics-of-north-america
